# Bolstering the adaptive information processing model: a narrative review

**DOI:** 10.3389/fpsyt.2024.1374274

**Published:** 2024-05-15

**Authors:** Jenny Ann Rydberg, Lisa Virgitti, Cyril Tarquinio

**Affiliations:** University of Lorraine, Inserm, INSPIIRE, Nancy, France

**Keywords:** EMDR, adaptive information processing, theory, psychotherapy, psychopathology, integration

## Abstract

In recent years, several theoretical models have been suggested as complementary to the adaptative information processing model of eye movement desensitization and reprocessing therapy. A narrative review of such models was conducted to assess the contributions of each, as well as their convergences, contradictions, and potential complementarity. Seven theoretical models were identified. All focus on the effects of EMDR therapy as a comprehensive psychotherapy approach with its principles, procedures, and protocols. Several refer to concepts related to propositional or predictive processing theories. Overall, the contribution of these proposals does appear to bolster Shapiro’s original AIP model, potentially offering additional depth and breadth to case conceptualization and treatment planning in clinical practice, as well as a more precise theoretical understanding. The current exploratory comparative analysis may serve as a preliminary baseline to guide research into the relative merit of suggested theoretical proposals to enhance current standards for the clinical practice and teaching of EMDR therapy.

## 1 Introduction

Considerable efforts have been deployed over the past three decades to explain *how* EMDR therapy works, i.e., how treatment effects are obtained on a neurological or cerebral level, by proposing different hypothesized mechanisms of action (1, 2). Most of these focus on the bilateral or dual attention stimulation (BL/DAS) component of EMDR, typically eye movements. However, the treatment outcomes of EMDR, a comprehensive psychotherapy approach, cannot be explained by BL/DAS alone; further mediators are believed to be found among the “core elements we believe are essential and unique to EMDR therapy in their aggregate form, as opposed to independent elements” (3, p. 192).

Theoretical models, on the other hand, primarily address the more abstract or higher-level question of *why* a treatment modality works. While they may subsume the issue of how certain mediators activate specific neurophysiological mechanisms, it is within a wider framework that may also address mental, cognitive, emotional, behavioral, somatic, and social levels. The purpose of such theoretical models of psychotherapy is to describe and explain the origin of psychopathology, the methods, techniques, and principles employed in the practice of a given approach, and the way they bring about therapeutic change.

The adaptive information processing (AIP) model is recognized by the vast majority of EMDR clinicians worldwide as unique, inherent, and essential to the practice of EMDR therapy (4, 5). Understandably, given this consensus, no alternative theories have been offered, although a small number of complementary theoretical models propose additional dimensions or constructs to expand Shapiro’s original model. To our knowledge, no review has yet been performed to identify, compare, or summarize these proposals, and they are very rarely cited by other EMDR-related literature. In other words, the potential value of their contributions remains largely untapped. The current paper represents a step towards bridging this gap.

## 2 Method

A narrative review methodology (6) was followed to identify articles presenting complementary, alternative, or contradictory theoretical models of EMDR therapy. To be included, articles needed to address Shapiro’s AIP model (4, 7, 8), while proposing novel theoretical hypotheses for the origin of psychopathology or EMDR treatment effects.

A comparative analysis was then conducted to explore how Shapiro’s AIP model may be both likened to and differentiated from other theoretical proposals.

The source for the original AIP model was the three editions of Francine Shapiro’s (4, 7, 8) seminal *Eye Movement Desensitization and Reprocessing: Basic protocols, principles, and procedures*. To identify articles addressing proposed additions to or modifications of the AIP model, two searches were performed for papers published until July 1, 2023: (1) a thorough inspection of all articles in the Journal of EMDR Practice and Research, and (2) a search on PsycInfo, Web of Science, and Google Scholar with keywords related to “EMDR,” “model,” “theory,” and “adaptive information processing”. From an initial identification of approximately 100 articles, eighteen were retained based on the title and abstract, and only nine remained after an in-depth exploration of their contents. These nine articles propose seven theoretical models (whereof two consist of two parts, thus two manuscripts).

The AIP model as well as of each of these seven other theoretical models are summarized below. Because of space restrictions and to allow for comparison, descriptions are abridged and thus necessarily incomplete. Authors’ recommendations for EMDR practice and discussions of neurological evidence have been considerably condensed. These descriptions are then completed by a comparative analysis.

## 3 Description of the theoretical models

### 3.1 The original model: adaptive information processing

The AIP model constitutes “the general model that provides the theoretical framework and principles” guiding EMDR treatment as well as “an explanation of the basis of pathology and personality development” (8, p. 30).

The AIP model is offered as a working hypothesis and posits that the brain includes an intrinsic, adaptive, physiological, information-processing system “configured to process … information and restore mental health in much the same way the rest of the body is geared physiologically to heal when injured” (7, p. 15). This system “allows information to be processed to an ‘adaptive resolution’ … the connections to appropriate associations are made and … the experience is … integrated into a positive emotional and cognitive schema … available for future use” (7, p. 29). Relevant to this hypothesis is the concept of memory networks, construed as associated systems or patterns of information, such as memories, thoughts, images, emotions, and sensations (8, p. 33; 4, p. 26). The term *neural network* was employed by Shapiro to refer to “the neurobiological configuration of an individual memory” (4, p. 26). EMDR therapy is conceptualized as progressing through memory networks associated with the initial treatment target—”a specific memory or dream image; a person; an actual, fantasized, or projected event; or some aspect of experience such as a body sensation or thought” (8, p. 34).

The information-processing paradigm provides a way to explain EMDR therapy’s “treatment effects and to guide the appropriate application of the method to a variety of presenting problems” (8, p. 16). When this information-processing system is blocked, dysfunction and pathology occur: most psychopathologies are “derived from earlier life experiences that set in motion a continued pattern of affect, behavior, cognitions, and consequent identity structures … (the) pathological structure is inherent within the static, insufficiently processed information stored at the time of the disturbing event” (7, p. 14). “[P]athology is viewed as configured by the impact of earlier experiences that are held in the brain in state-specific form” (4, p. 15).

Shapiro’s initial discovery, during her walk in the park in 1987, enabled the understanding that rapid eye movements foster the accelerated information processing of the past experiences that underlie current dysfunction and pathology. It was subsequently shown that other forms of BL/DAS (auditory or tactile) may have similar effects. Clinical experience and feedback led to the development and refinement of principles, protocols, and procedures consistent with the AIP model, which, applied as a comprehensive treatment approach, result in “greater treatment effects than those produced by the initially described EMD” procedure (4, p. 15), generating not only a *desensitization* effect (i.e., “a reduction in emotional or physical reactivity to stimuli that is achieved by such means as deconditioning techniques”; 9) but also the cognitive restructuring of memories, the elicitation of spontaneous insights, and an increase in self-efficacy, named *reprocessing* (8, p. 13).

Shapiro proposed the AIP model as a unifying theory underlying all psychological modalities (7, p. 52) since they all “can be defined as ultimately working with information stored physiologically in the brain” (p. 17). Thus, EMDR therapy may be considered as one method among “a set of Accelerated Information Processing treatments” (p. 29).

### 3.2 Complementary models

#### 3.2.1 A dialectical perspective

Dialectical constructs are constructs of polarity resulting in a dynamic unity of opposites. Laub and colleagues’ dialectical perspective (10–12) is presented as applicable to various psychotherapeutic approaches, with the potential of enriching the understanding of how the adaptive information-processing system functions and how to facilitate it.

This view is built on the dialectical premise that change stems from the inherent motion of a developmental process toward optimal integration—more specifically, two information-processing movements: the horizontal dialectical movement between opposites and the vertical dialectical movement of whole/part shifts.

The horizontal dialectical movement can be likened to Shapiro’s (8) proposed linkage of dysfunctionally stored and adaptive information through their respective memory networks in EMDR therapy. Laub et al. (10) also describe it as the motion of the emerging sequence of thesis, antithesis, and synthesis, this last step representing a higher level of integration with a new balance.

The vertical dialectical movement rests on the observation that the universe functions like a greatly differentiated ensemble of interacting systems, organized hierarchically within larger systems: a whole thus becomes a part of a higher whole. This hierarchical organization expands through whole/part shifts towards greater integration or wholeness. Such a sequence can proceed from a fragment to an event, then to an episode, a theme, and, finally, an identity.

This dialectical perspective, with its two movements, illustrates how differentiation and linking constitute complementary aspects of the AIP system. Psychopathology such as PTSD arises when there is excessive differentiation (avoidance, hypoactivation) or excessive linkage (intrusions, hyperactivation). EMDR therapy facilitates the restoration of balance within the two dialectical movements, the integration of experience, as well as the creation of new adaptive memory networks.

In clinical practice, this perspective entails a focus on facilitating the horizontal dialectical movement between opposites (e.g., between the traumatic memory and a resourced experience, between past dysfunctional relationships and the current therapeutic alliance) and the vertical movement of whole/part shifts for the integration of the many aspects of experience.

#### 3.2.2 The theory of neural cognition

The theory of neural cognition (or *Théorie neuronale de la Cognition*: TnC) is a general framework that aims to elucidate cognitive processes at the neural network level (13, 14). Khalfa and Touzet (15) argue that EMDR therapy’s treatment effects can be explained simply by the properties of normally functioning neurons and neural networks.

In the TnC, “the cortical column is the unit of information processing [that codes] continuous values, [whereas a single] neuron only [codes] transient binary values” (13, p. 2). A cortical column is a functional set of 110,000 neurons. Such cortical columns of neurons are necessary to ensure sustained neural activity since a single neuron will be depleted after a few dozen repeated depolarizations (spikes).

“The total number of brain neurons is estimated to 82 billion, but the cortex accounts only for 20% of the total number of neurons in the brain. It follows that the number of cortical columns is close to 160,000. Careful recent analysis of the cortical architecture has shown that the cortex is [composed] of 360 areas (or cortical maps)” (13, p. 2) with an average of 450 columns per map. “Each map is [dedicated] to a specific dimension of [an] event. Cortical maps receive the sensory inputs form the visual, auditory, olfactory, and proprioceptive cortices (or primary cortex). The secondary cortex [comprises] the maps that receive inputs from the primary cortex’ maps, [establishing] associations such as between form and color”.

Research has identified the functional role of eighty of the above-mentioned 360 cortical maps, which consists of coding for a certain dimension of reality (i.e., a high-level representation, such as machines, faces, body parts, animals). The cortex can thus be understood as “a hierarchy of maps, each [map] coding for [a] specific dimension of a situation or event, and each map organized” (13, p. 2) in accordance with the person’s singular experiences. “On a given map, at any time, due to local inhibition between columns, there is an inter-column competition, each [map] inhibiting the others, but also being inhibited by them. [Within the entire] hierarchy of cortical maps, only a small number of columns are fully activated at any one time,” depending on the specific situation. “These activated columns form a sparse coding representation of the situation: the global state of activation (GSA)”.

Memories are traces of experienced events (GSAs) that enable the recognition of a present event as either highly similar to a past situation (i.e., an existing GSA), partially similar for certain aspects (i.e., a partly similar GSA), or largely dissimilar from everything that the person has already encountered (i.e., no correspondence with any GSA). If the individual experiences an event that is identical or highly similar to one that has been previously memorized, there is a strong possibility that what will occur next will also be highly similar to what followed the same event when it was experienced in the past.

Cortical maps function in a manner consistent with their topology: comparable information inputs will activate columns that are close to each other, whereas very different inputs will activate columns that are more distant from each other. The activation of neighboring columns following the identification of a particular dimension of an event facilitates the prediction of the future value of the next event in this dimension. When all maps in the cortical hierarchy are considered, the brain makes predictions and initiates appropriate and corresponding actions.

Another concept that is important in the TnC is that of long-term potentiation (LTP), described as intervening “to enhance the matching between the activated cortical columns, strengthening their connections’ efficacy. At the same time, long-term depression (LTD) erodes the connections between columns that are not activated. This synergy between LTP, LTD, and the cortical neural architecture is sufficient to organize the maps’ hierarchy and to achieve precise representations of all experienced situations” (13, p. 3).

Since LTP and LTD are not limited to cortical neurons, all neurons providing input to the cortex and all neurons receiving output from the cortex also experience synaptic efficiency adjustments according to their use. In the same vein, GSAs are not “restricted to the cortex, but [concern] neurons [of] all brain structures, such as the hippocampus, thalamus, amygdala, etc. All neural activities of a GSA are coherent, i.e., they are part of an attractor [that] bends the activities’ dynamics towards the memorized GSA” (13, p. 3).

##### 3.2.2.1 Theory of neural cognition, stress, and traumatic memory

Physiological modifications associated with potentially dangerous situations are known as *stress*. These modifications are adaptive solutions, which include improved strength and accelerated processing. A potentially dangerous situation must be recognized and identified as such as quickly as possible, for the shortest reaction delay possible. This delay increases by 10 ms at each cortical step. Therefore, the requirement for speed wins over the need for precision: recognition is achieved in a single cortical step, even if it is cruder. That is the role of the amygdala, which acts as an early warning system that initiates fight-or-flight behaviors. In parallel, the cortex analyses the situation in detail and may either interrupt the initiated defensive behavior (in case of a false alert) or complete the action underway (in the event of a confirmed threat).

The amygdala is known as essential “in the acquisition and expression of fear conditioning, as well as its extinction. It has strong connections to the medial prefrontal cortex (mPFC)” (13, p. 4).

The accelerated processing resulting from stress entails an improved neural memorization, whereby a single occurrence allows for learning, automatically reinforcing the GSA associated with the stressful situation. This GSA will easily be recalled, and each recall will generate the same physiological stress response, leading this GSA to prevail over all others. This corresponds to acute stress disorder or PTSD.

The stressful situation experienced by the person leads to the formation of a traumatic memory, a part of episodic memory. When compared to controls, in PTSD patients, such situations appear to entail an abnormal activation of the amygdala and the prefrontal cortex. The amygdala recognizes the traumatic event, which is also processed by the cortex. “Synaptic LTP guarantees that the memorization of the traumatic event implies neural connections between the amygdalae and the cortex … The GSA of the traumatic event includes an activation of the amygdalae in addition to its cortical representation. Each time the event is recalled … a part of the amygdalae is also activated [with] its automatic stress response … inducing … a negative emotion. Each recall reinforces the association between the cortex and amygdalae” (13, p. 4).

In PTSD, flashbacks can be understood as repeated recalls of the traumatic events that maintain, or even reinforce, the stress response. However, a potentially traumatizing event does not systematically lead to PTSD. “According to the TnC, the … event’s effect will depend upon pre-existing cortical and amygdala connections for similar GSAs. The more a set of GSAs have been reinforced by several traumatic or deleterious events, the more a person could be at risk for developing PTSD” (13, p. 4).

##### 3.2.2.2 Bilateral or dual attention stimulation and new global states of activation

In the TnC, Khalfa and Touzet (13) relate BL/DAS to GSAs in the following manner: sensory neurons perceive the BL/DAS and relay the information to their target neurons, which propagate the information, and so forth. When the therapist elicits the client’s traumatic memory, the current GSA is the one representing the traumatic memory (GSA0). Further information is progressively developed, leading to the addition of new column activations to GSA0. The new GSA—GSA1—is a stable GSA, i.e., one for which the added columns are relevant, and this added information can be verbalized by the client.

GSA1 is larger than GSA0, and Khalfa and Touzet (13) specify that it does not involve any new connections with the amygdala. The amygdala activation decreases in weight in comparison to the cortical activation. During each iteration of trauma recall and BL/DAS, new column activities are added to the current GSA. After n BL/DAS sets, the initial GSA0 has been replaced by a new, larger, GSA—GSAn—which no longer elicits the amygdala given the absence of any connections in the additional columns. In addition, the prefrontal cortex is more involved in the new GSAn. The stress response no longer takes place and the client no longer experiences intense negative emotions related to the traumatic memory. The additional columns correspond to aspects that are new in the context of GSA0 and this can be described as *memory reconsolidation* or the learning of new associations.

Finally, Khalfa and Touzet (13, p. 5) argue that TnC can also explain “why [bilateral alternated stimulations] are more efficient than bilateral non-alternated stimulations or unilateral ones. Bilateral stimuli have a largest recruitment area compared to unilateral stimuli. The ability of the stimulations to recruit a large area depends upon predictions at map level that elicit inhibition processes. Alternation and intermittency are discontinuities that do not favor predictions. Since predictions authorize inhibition, less predictable [bilateral alternated stimulations] are more efficient than bilateral non alternated or bilateral non intermittent stimuli”.

Regarding the AIP model, the TnC agrees that traumatic events are stored in the brain with their original emotions, sensations, and beliefs, and are later reconsolidated. The TnC’s explanations address the underlying neural mechanisms of reconsolidation and argue that this memory reconsolidation is “both learning of new associations, and forgetting of old ones” (13, p. 6). However, no specific recommendations for clinical practice are provided.

#### 3.2.3 The three-dimensional model of experiential selfhood

Fingelkurts and Fingelkurts (16) suggest that their previously published, neurophysiologically-based, three-dimensional construct model of complex experiential selfhood (17, 18) may be applied as a more comprehensive theoretical model of EMDR therapy, since other hypothesized mechanisms of action, which they group into three broad classes (working memory, psychophysiological, and sleep-related) fall short of explaining the totality of the effects of EMDR therapy in the treatment of posttraumatic stress disorder (PTSD).

The three-dimensional model of experiential selfhood (3DMES) is based on the neurophysiology of the default mode network, described as the self-referential brain network, and on the functional-topographical specialization of three subnets or operational modules within this network, as studied both under normal, healthy conditions and during pathological conditions with diminished or lost self-consciousness.

The three brain operational modules (OMs; the anterior OM, the right posterior OM, and the left posterior OM) can be easily and reliably estimated by applying operational analysis to the EEG signal. They denote three different types of self-referential processing, which together construct a unified sense of self.

The *anterior module* mediates the first-person perspective and sense of agency. It can be likened to the ‘witnessing observer’ or the sense of ‘Self’. The *right posterior module* supports (a) the experience of self as a localized, embodied entity, through interoceptive and exteroceptive processing, (b) emotion-related thoughts, and (c) autobiographical memories, which, together, underlie representational-emotional agency or the sense of ‘Me’. The *left posterior module* accompanies the experience of thinking about and reflecting upon oneself, including momentary narrative thoughts and inner speech. This refers to reflective agency or the sense of ‘I’.

Each module is irreducible into the others; it can be enhanced or weakened following the individual’s physiological and mental state, training (e.g., meditation), or pathology (e.g., PTSD).

The Fingelkurts brothers state that their research examining functional integrity (by means of EEG operational synchrony) shows that individuals with PTSD symptoms exhibit a pattern with increased integrity of the anterior OM (‘Self’ component) and increased integrity of the right posterior OM (‘Me’ component) alongside with decreased integrity of the left posterior OM (‘I’ component). These results help to explain the experience of individuals with PTSD: hyperactivity, enhanced vigilance to self and surroundings = increased ‘Self’; enhanced emotional, sensory, and somatic states that tend to reoccur as persistent intrusions = increased ‘Me’; and greater avoidance/decreased narration, verbal representation, and lack of linguistic/contextual information, often leading to detachment and depersonalization/derealization = decreased ‘I’ (16).

These results lead to recommendations that therapy for PTSD should aim to increase functional synchrony within the left posterior OM and to decrease functional synchrony within the anterior and right posterior OM. Such changes correspond to the effects of EMDR therapy. While such effects may be observed with other psychotherapy approaches as well, Fingelkurts and Fingelkurts argue that EMDR therapy is uniquely suited to fulfill these goals because of the neurophysiology of eye movements (EMs). Their detailed explanation refers to saccadic eye movements, with the finding that EM-related neural activity changes predominantly occur within the alpha frequency range, which corresponds to 7–13 Hz oscillations in the EEG signal. The authors provide no recommendations or implications for clinical practice.

#### 3.2.4 The network balance model of trauma and resolution 

Chamberlin’s (19, 20) network balance model of trauma and resolution (NBMTR) aims to clarify the biological basis of how the dysfunctionally stored memories postulated by the AIP model are created, then resolved to a state of mental health, using EMDR therapy as an example.

##### 3.2.4.1 Level I

NBMTR’s *first level* (19) is based on the triple network model of psychopathology, which considers that the major clinical syndromes can result from dysfunction of the brain’s large-scale neural networks (21): the default mode network (DMN; responsible for internal mentation), the central executive network (CEN; active when a subject engages in a task with the external world), and the salience network (SN; the “network switch” involved in emotional processing, homeostatic regulation, and reward). While the optimal processing of experience requires the coordination of these networks, this balance can be compromised or lost under conditions of severe stress, impeding the coordination between critical structures embedded in these networks (e.g., hippocampus, amygdala, and prefrontal cortex).

While the triple network model and its applications to PTSD emphasize dysfunction within individual networks, the NBMTR posits that dysfunction arises from the disruption in patterns of interaction between the large-scale networks as a complex adaptive system. PTSD results from inadequately processed and dysfunctionally stored memories and the accompanying failure to restore network balance. Therefore, the critical factor in PTSD treatment is the restoration of network balance and adaptive information processing, combining emotional processing (SN), the elaboration of associated cognitions (CEN), enabling awareness of inner experience (DMN) as well as the external environment (CEN).

The NBMTR describes how, in PTSD, the DMN is hypoactive (since it corresponds to the prefrontal cortex and hippocampal areas), as is the CEN (also related to the prefrontal cortex), while the SN is hyperactive (related to the amygdala). Furthermore, in PTSD, the disruptions in network balance caused by stress do not resolve, leading to a vicious circle: the functions of the prefrontal cortex are reduced, the amygdala then produces even more norepinephrine and dopamine, further reducing the activity of the prefrontal cortex, which over time may result in a state of lasting dysregulation, impeded information processing, and the formation of memories that are characterized by vivid ‘flashbulb’ memories, with amnesia for contextual details, and fragmentation – the ‘dysfunctionally stored memories’ of the AIP model. It is precisely this vicious circle that maintains network imbalance and blocks the AIP system, resulting in dysfunctional processing of certain memories.

From the NBMTR perspective, EMDR therapy’s protocols and procedures are particularly well suited for eliciting and promoting the balance of large-scale neural networks. Chamberlin argues that the standard protocol is highly compatible with what he calls ‘contemporary network science,’ each phase activating specific neural networks in a particular order. Therefore, no new modifications to current protocols and procedures are suggested. The EMDR therapist facilitates a state of network balance that is necessary for adaptive information processing of memories. Interventions for blocked processing include changing direction or speed of eye movements (activating the CEN), attending to sensations (activating the SN), or returning to target (activating the DMN), to reestablish network balance. From the perspective of the NBMTR, if the networks are balanced, the memory will process.

##### 3.2.4.2 Level II

NBMTR’s *second level* (20) focuses on the role of memory as the principal substrate for predictions that guide behavior. It can be described as a goal-directed processing perspective, which postulates that if the networks are balanced, poor predictions based on dysfunctionally stored memories will be mismatched and the memories updated.

Predictive processing theory allows for the understanding of many cognitive activities, such as perception, attention, learning, from the perspective that the brain’s main function is to predict its own immediate experience through probabilistic inference, to use sensations as feedback to verify the accuracy of its predictions, and to minimize prediction errors.

The predictive processing model of EMDR focuses on memory as the principal substrate for predictions that guide behavior through cycles of perceptual inference. Incoming sensory information cues the retrieval of specific memories. The brain alternates searching the external world with searching memory in a constant flow of processing. This cycle of selecting incoming information, matching from memory, predicting, and further sampling continues throughout life, as the brain attempts to minimize the errors of its predictions. When the brain registers a prediction error, it may update the memory through a memory reconsolidation process, thus reducing uncertainty and ensuring more successful behavior in the future.

The predictive processing perspective is highly compatible with the AIP model’s description of dysfunctionally stored memories as the foundation of posttraumatic psychopathology. If memory is the substrate of predictive processing, then such state-specific memories, frozen in time, will generate prediction errors and suboptimal behavior. The NBMTR postulates that imbalance of the SN, DMN, and CEN compromises the coordinated interaction of brain regions required to execute this processing, impeding the brain’s habitual actions to minimize its prediction errors and improve future predictions and behavior. Once network balance is restored, memory will be processed and reconsolidated. No recommendations for clinical practice are formulated at this level.

Chamberlin extensively explores research data on eye movements as they relate to predictive processing, to explain several clinical phenomena observed in EMDR therapy, such as restoring attention, facilitating memory search, and amplifying prediction errors to enhance memory reconsolidation. His detailed exploration of neurobiological research refers to findings suggesting that “the hippocampal theta rhythm is crucial in organizing the flow of information through the neural circuits responsible for the encoding and retrieval of episodic memory … saccadic eye movements play a critical role in this regard by resetting the theta rhythm and thus synchronizing the flow of incoming information through disparate regions including the hippocampus and prefrontal cortex in processing experience and memory” (20, p. 5).

#### 3.2.5 The Zeigarnik effect

The Zeigarnik effect (ZE) is a property of memory discovered in 1927 by psychologist Bluma Zeigarnik, who observed that individuals have better recollection for interrupted tasks in comparison to completed ones. Fox (22) shows that several of EMDR’s treatment components contain ZE-related mechanisms that may contribute to EMDR therapy’s effectiveness and efficiency.

The ZE directs attention to the unfinished goal, notably via intrusive memories and the binding of cognitive resources. The failure to complete personally meaningful tasks drives the motivational component of rumination, generating a memory bias toward completion of interrupted behavior.

This lack of completion is characteristic of traumatizing events—thus, ZE is implicated in the development and maintenance of PTSD. From an AIP perspective, the ZE is related to intrusions, ruminations, and reexperiencing characteristic of PTSD, which can be understood as attempts to integrate maladaptive memory networks of unprocessed traumatic experiences into adaptive memory networks. On the one hand, rumination may facilitate integration, but on the other, it may also amplify subjective discomfort and result in overwhelm or subsequent avoidance. Dual demands to both assimilate and avoid traumatic material are responsible for the hyperarousal/reexperiencing and avoidance observed in PTSD.

Fox argues that activation of the targeted memory network in EMDR therapy is sustained by the ZE drive for completion, eliciting impulses toward the resolution of incomplete actions and thus strengthening motivation in the following phases of the treatment process.

Prospective memory, which allows a person to remember to carry out intended actions at a given time in the future, entails both a prospective dimension (the recollection of memory at the appropriate time) and a retrospective dimension (the remembrance of the task itself). Individuals often recognize that a traumatic event is incomplete but fail to act on prospective intent. EMDR may alleviate both the retrospective and the prospective aspects of prospective memory through the different phases and prongs of the standard protocol.

During EMDR processing, the client is repeatedly interrupted in their focus on the memory by the question, “What do you get now?” These interruptions are likely to heighten the ZE, by adding salience to memory of the unfinished task (the traumatic event), thus sustaining attention and motivation toward the completion of action. In terms of clinical implications, Fox recommends that therapists identify events experienced as unfinished for targeting and encourage clients to imagine how they would like such situations to end, to increase their motivation toward completion.

#### 3.2.6 The biopsychosocial adaptive information processing model

Cotraccia’s biopsychosocial AIP (BPS-AIP) model (23) expands on the psychological and social dimensions in addition to Shapiro’s description of neural networks as inherent to the information-processing system. The effects of adverse life experiences on the AIP system are viewed not only as disruptions of neurophysiological structures, but also as imbalances in personal and interpersonal processes of communication and representation.

In this view, attachment relationships may either provide the context of trauma or facilitate the access to adaptive information (past states of adaptive actions) and the appropriate update of self- and world models (the source of adaptive resolution or healing). The pathogenic nature of traumatic experiences lies in their capacity to disrupt communication and representation at the subpersonal (brain), personal (self), and interpersonal (others, relationships) levels.

For Cotraccia, the biopsychosocial availability of adaptive information is essential to the efficacy of EMDR therapy. Early relationships, with their examples of communicating with caregivers and constructing adaptive ways to deal with stressful situations, constitute context-sensitive constraints that structure experiences into either healthy/integrated or segregated/disintegrated inner working models (IWMs). Each IWM can be seen as an attractor, i.e., the state space a system will return to after a momentary disturbance.

From the perspective of information-processing theory, BPS-AIP conceptualizes trauma as disruptive noise—it is defined more by the lack of resources for attunement and communication than by the nature of the stimuli present in the experience. In contrast, the capacity for intrapersonal and interpersonal attunement serves as an indicator of the robustness of the BPS-AIP system. In EMDR therapy, the psychosocial components of the therapeutic relationship are seen as causally related to positive outcomes, through the provision of a context enabling the consolidation process of autobiographical memory.

Ten years after his initial publication, Cotraccia (24) expanded his model to incorporate Graziano’s (25, 26) work on attention schemas and social cognition, defining an attention schema as content integrated with implicit self-models that maintain subjective mental states of BPS connectivity or disconnectivity. According to this theory, the brain contains a model or schema of itself paying attention and predicting what it and others will pay attention to.

In a connected BPS-AIP system, there is a degree of integration between the subpersonal, personal, and interpersonal levels that enables the individual to maintain autonoetic consciousness (experience of self) under stress and over time. In a disconnected BPS-AIP system, however, there is a likelihood of losing one’s experience of autonoetic consciousness under stress.

Stressful life experiences become traumatizing when there is a failure of global BPS-AIP connectivity. Subsequently, the BPS-AIP system reorganizes around the lack (absence) of attentional resources: there is an intolerance for maintaining attention on one’s self-process, and the scarcity of information collected from subjective experience impedes self-regulatory and homeostatic functioning. A BPS-AIP system that organizes itself around disconnection is constantly seeking something that is not there, but ought to be present. The person’s behavior and attention are focused on the experience of others and leave the trauma unattended.

The therapeutic relationship in EMDR therapy offers that which ought to be present, but that has been missing: a self-modeling system that enables the adjustable tracking of attention between therapist and client within an interpersonal interaction; this, in turn, supports the client in attending to their self-process.

In terms of implications for clinical practice, Cotraccia recommends identifying maladaptive attractors or IWMs to target these relational experiences and representations of self and the world. He also underscores the value of the therapeutic relationship and reparative attachment experiences, as well as the importance of enhancing autonoetic consciousness, in producing treatment effects.

#### 3.2.7 A goal-directed predictive processing perspective

Vanderschoot and Van Dessel (27) discuss recent evidence that contrasts with dominant theories of fear, anxiety, and stress-related disorders in general, and PTSD specifically, as well as with theories of trauma-focused therapies, including the AIP model, which traditionally draw on conditioning effects and associative mental processes. Propositional theories, on the other hand, argue that the production and activation of propositional information (inference-making) fosters and supports maladaptive behavior.

As these authors point out, neuroscientific insights in recent years have contributed to the increased popularity of predictive processing (PP) theories, which predicate that belief-based processes involving causal inferences (i.e., predictions) underpin cognition.

Propositional information differs from associative information in that it has a truth value and can encode variations in the type of relation between two events or representations (e.g., “speaking up can *protect against* rejection” or “speaking up can *cause* rejection”) rather than merely specifying a link between them (e.g., the link between “speaking up” and “rejection”). Thus, in contrast to associations, which cannot capture beliefs, propositions support inferential reasoning. These differences come with important implications, supporting the idea that propositional theories of PTSD may bring added value in comparison to associative theories.

Vanderschoot and Van Dessel demonstrate how the AIP model can be adapted to integrate lessons from propositional theories. They argue that while some authors like Chamberlin (20) have attempted to explain PTSD and EMDR treatment effects within the PP framework, “these theories focus on explanation at the neural level rather than [the] behavioral level, and therefore do not provide guidance to predict and influence behavior that can be readily integrated into clear recommendations for clinical practice … PP theories [comprise] many different implementations and often involve reference to several complex constructs and processes” (27, p. 112) that can prove difficult to translate and integrate into EMDR theory and practice. Therefore, their goal-directed predictive processing (GDPP) perspective identifies key premises of prominent propositional theories at the cognitive and mental process level.

##### 3.2.7.1 Key premises

*The mental system as a network of beliefs about the world.* Inferences are drawn from these beliefs and underlie thoughts, emotion, and behavior. Causal inferences or predictions influence perceptions, whereas behavior corresponds to ‘active inference’ that involves predicting one’s own behavior.

*Highly automatic inferences that follow general principles of biological systems such as entropy reduction.* An individual’s belief network consists of different belief modules, activated by specific contextual stimuli, which evoke predictions. “These modules have a hierarchical structure such that higher hierarchical levels contain more generative beliefs (i.e., beliefs that generate more predictions and are more generally applicable), whereas lower hierarchical levels contain beliefs that are only applicable to certain situations or aspects of the world” (27, p. 112). Beliefs from higher hierarchical levels have more weight and can thus overrule beliefs from lower levels. The goal of minimizing the disorder generated by prediction errors underlies the process that updates beliefs and assigns them higher or lower generative power.

*Context-dependent inferences about desired outcomes (i.e., goals)*. Beliefs about wanted outcomes lead to inferences about actions and behavior likely to achieve these outcomes. In this view, goals determine all behavior. The activation of beliefs about a given, contextually activated, desired outcome can generate inferences that lead to maladaptive behavior that may conflict with other personally relevant goals.

In summary, this GDPP “explains behavior (and thoughts and emotions) as the result of three inference steps. First, internal or external cues lead to the registration of (homeostatic) wanted states (i.e., goals) … Second, to reduce prediction error between wanted and actual states, inferences are made about the outcomes of contextually relevant actions … Finally, given a sufficient match between predicted action outcomes and current goals, one predicts engaging in the action and the action is elicited” (27, p. 113).

##### 3.2.7.2 A goal-directed predictive processing perspective on PTSD

The AIP model considers that traumatic memories are stored in distinct memory networks (belief modules in PP), unconnected to the adaptive information contained in other networks. In the GDPP perspective, a traumatic event may evoke a significant prediction error because of the unforeseen vast discrepancy between the current (unsafe) state and the expected (safe) state. This prediction error is attributed a strong value because it conflicts with the aim to be safe and to survive (a key homeostatic objective represented at a very high level). To minimize prediction error in case this unexpected state were to occur once again, the belief network is instantly updated. However, the event may not be integrated within other present belief modules because of the discrepancies with highly generative beliefs. Instead, a new module may be formed, integrating as much sensory information as possible to provide ample opportunity to update the belief module in the future.

In contrast to the assertion that memories and beliefs about the traumatic event are frozen in time or stored in a state-specific form, the GDPP perspective considers that it is unlikely that processing is suspended in a prolonged manner (as there would be high entropy in the general belief network). Rather, there will be repeated attempts to integrate traumatic event memories into current belief networks. When trauma-related stimuli facilitate the prediction of similar events, prediction error will ensue, because the event is not encountered once more, and the relevant beliefs and predictions will lose their influence. In other words, these beliefs and predictions are represented at a lower level, which entails that they will be activated in fewer contexts and have a weaker impact on behavioral prediction.

In individuals at risk for PTSD, however, predictions may not be updated in this way. Instead, they may believe, maladaptively, that unpleasant events are likely to occur and that it is only their avoidance behavior that prevents the recurrence of these events. Consequently, they may continue to avoid the feared situation, thereby preventing any adaptive updating of their predictions. From this perspective, the generative beliefs available in a person’s belief network determine why some individuals do and others do not develop PTSD.

##### 3.2.7.3 A goal-directed predictive processing perspective on EMDR therapy

The GDPP perspective is highly compatible with the AIP model in the sense that EMDR therapy is understood as promoting the integration of information stored in trauma-related memory networks with information from more adaptive networks. A major difference, which might be considered as a valuable update to the AIP model, is that relevant beliefs and predictions (rather than associations) should be seen as the main target of therapy (placing the focus on the updating of beliefs rather than on fostering associations). More precisely, changes arise because of prediction errors that facilitate the integration of traumatic and adaptive information.

For optimal effectiveness, therapy should then focus on supporting clients in learning to predict a reduction in their symptoms and represent these predictions at higher hierarchical levels to foster changes in behavior outside of the therapeutic context. Clients should also be encouraged to confront avoided situations to elicit prediction errors, as well as supported in forming new inferences based on past experiences of success and adaptive behavior. In the GDPP perspective, the key determinant of treatment success is the extent to which a client learns to predict EMDR treatment efficacy, based on their initial sessions of reprocessing a target that had been thus far avoided, experiencing a reduction of the associated disturbance and a modification of their related beliefs/predictions (e.g., from “I am in danger” to “I am safe”). These initial successes (the experience of processing a disturbing memory to its adaptive resolution) lead the client to predict the success and efficacy of EMDR therapy in the treatment of further (past, present, future) targets.

## 4 Comparison of the theoretical models

The comparative analysis of the original and complementary theoretical models, included in this review, focuses on the following questions:

- On what level(s) do explanations or hypotheses focus (neural/neurophysiological, mental/cognitive, behavioral, etc.)?
- If included, what type of neurological evidence is portrayed?
- Is there an explanation of psychopathology and does it relate to PTSD specifically, to trauma-related pathology or dysfunction, or to psychopathology and personality development in general?
- Are EMDR treatment effects attributed to or explained by BL/DAS?
- Are other potential mediators or simply the general EMDR principles and procedures mentioned as also responsible for treatment effects?
- Is the AIP system as an innate system of the brain mentioned?
- Is the AIP model addressed?
- For the complementary models, what are the novel aspects or constructs? Specifically, is the model based on associative or predictive processing?

### 4.1 Adaptive information processing model

Shapiro’s original AIP model (7) addresses the mental and behavioral levels, while stipulating that these translate to the neural or neurophysiological level. It was developed before sufficient neurobiological data was available, based on clinical practice-based evidence. Successive editions of Shapiro’s seminal book (4, 8) incorporate the latest available research data into the original model without bringing about any fundamental changes. The AIP model aims to explain the origin of all psychopathology (that is not biologically based or chemically induced) and of personality development, which are understood as deriving from the insufficiently processed, maladaptive, traumatic memories that are held in a state-specific form. Treatment effects are attributed not only to BL/DAS but to the entire comprehensive psychotherapy approach with its principles, procedures, and protocols. The central tenet of the AIP model corresponds to the existence of an intrinsic information-processing system, in which associative processing plays a major role, while considering that EMDR therapy constitutes a particularly effective and efficient way to restore and enhance the functioning of this innate system; this effect is described as accelerated information processing.

### 4.2 The dialectical perspective

The dialectical perspective (10) is situated on the mental and philosophical levels. It does not rely on neurological evidence. Its explanation of psychopathology rests on the impediment of the two dialectical movements, illustrated through the example of PTSD, without elaborating on how this process might foster other forms of psychopathology or dysfunction. The model does not focus on the specific effects of BL/DAS, but rather considers the principles and procedural steps of EMDR therapy. It refers to associative processing and does not contradict nor address any potential limits of the AIP model, but merely proposes an explanation of how the innate AIP system functions, what impedes it, and how EMDR therapy restores its functioning in a manner compatible with general principles of therapeutic change.

### 4.3 The theory of neural cognition

The TnC (13) addresses the neural level, and how it translates to the cognitive level, to provide an original description of the innate AIP system. It provides ample detail regarding the organization of neurons into cortical columns and maps, their functioning, as well as the formation of GSAs throughout all brain structures. Its explanation of psychopathology is limited to PTSD. EMDR treatment effects are related both to BL/DAS and to EMDR procedural steps in general. The model refers equally to associations and to predictions, without raising any potential contradictions between the two types of processes. Regarding the AIP model, the TnC agrees with the notion that traumatic events are stored with their original emotions, physical sensations, and beliefs, and that they are reconsolidated with EMDR therapy. According to this view, memory reconsolidation is both learning of new associations and forgetting of old ones, and this learning is related to the predictive role of the GSAs of neurons in cortical columns and other brain structures. In summary, while this theory expands on Shapiro’s AIP model, it does not argue in favor of any corrections or modifications, nor does it address the effectiveness of EMDR therapy beyond the treatment of PTSD.

### 4.4 The three-dimensional model of experiential selfhood 

The 3DMES (16) is situated on the neural level and focuses specifically on the DMN. It does not attempt to explain psychopathology or personality development in general, merely focusing on PTSD. Based on this disorder, it explains both the origin of the pathology and how psychotherapeutic change is obtained. Some concepts, such as the differences between what the authors call Self/Me/I, would have merited further clarification. The Fingelkurts brothers claim to offer a more comprehensive theoretical model to explain the totality of EMDR treatment effects and of psychotherapeutic change overall, while developing the reasons why they believe that the saccadic eye movements of EMDR therapy may be particularly effective in producing such therapeutic change. The AIP as an innate system is not addressed, and the AIP model is only briefly mentioned to state that it does not provide a satisfactory explanation at the neurobiological or neurophysiological level. Lastly, this model does not reason in terms of either associations or predictions, nor does it argue in favor of any modifications to Shapiro’s AIP model.

### 4.5 The network balance model of trauma and resolution 

The NBMTR (19, 20) mainly considers the neural level and its repercussions on the cognitive and behavioral levels. Its neural starting point is the concept of the necessity of balance between the three major large-scale neural networks for mental health and optimal functioning. It further explores the cognitive dimension through a predictive processing perspective. Hypotheses on the model’s first level concern psychopathology in general, while the model’s second level pays particular attention to PTSD in relation to the maladaptive, frozen-in-time memories described by Shapiro. Effects are not limited to BL/DAS. The NBMTR predictive processing model of EMDR focuses on memory as the principal substrate for predictions that guide behavior through cycles of perceptual inference. However, the NBMTR does also mention associative processing (e.g., referring to “associations linking disparate networks”), without addressing any potential discrepancies between associative and predictive processes. It is presented as coherent and compatible with the AIP model, and thus does not propose any changes to Shapiro’s model.

### 4.6 The Zeigarnik effect 

The ZE (22) is located on the mental and cognitive level. While it does not rely on neurological evidence, it does attempt to provide neural and psychophysiological data that are compatible with its hypotheses. Psychopathology is conceptualized through the lens of trauma; personality development is not addressed. BL/DAS is seen as only one mediator among many associated with EMDR therapy’s procedural steps. It addresses the innate AIP system as a system of memory reconsolidation and refers to the AIP model. It does not aim to criticize or contradict Shapiro’s model, but simply offers an additional explanatory cognitive mechanism. Referring to both associations and predictive processing, it does not address any potential contradictions between the two.

### 4.7 The biopsychosocial adaptive information processing model

The BPS-AIP model (23, 24) focuses on the cognitive and social/interpersonal levels through the lenses of trauma as noise and trauma as absence, to explain the development of personality and psychopathology (without using those terms specifically). While eye movements are mentioned, they are presented as only one mediator among many that explain the treatment effects of EMDR therapy. The BPS-AIP model suggests that the innate AIP system is inherently a biopsychosocial system. It relies on attention schema and predictive processing theories to bridge the gap between neurophysiological hypotheses regarding mechanisms of action and the impact of interpersonal experiences, involving attachment and attunement, or their insufficiency. Both associations and predictions are mentioned, without contrasting the two types of processes. BPS-AIP is presented as a proposal to complete rather than change Shapiro’s AIP model.

### 4.8 A goal-directed predictive processing perspective 

The GDPP perspective (27) focuses on the mental and cognitive level. It provides an explanation for the development of PTSD and for the treatment effects of EMDR therapy for PTSD, without envisaging other forms of psychopathology or personality development. Eye movements or other forms of BL/DAS are understood as playing an important role, but not solely responsible for outcomes. Indeed, the extent to which a client learns to predict EMDR treatment efficacy is seen as decisive. Shapiro’s hypothesized AIP system is not addressed as such, but the GDPP perspective considers that the mental system constitutes a network of beliefs about the world. While agreeing with the AIP model in that EMDR therapy is understood as enabling the integration of trauma-related information with more adaptive information, Vanderschoot and Van Dessel advocate for an important modification to the AIP model, whereby the main target of therapy would correspond to relevant beliefs and predictions (rather than associations), placing the focus on the updating of beliefs rather than on the fostering of associations. In this view, prediction errors promote change by facilitating the integration of traumatic information with adaptive information that is sufficiently consistent with the client’s belief network.

## 5 Discussion

Seven theoretical models were identified and described in this narrative review. While their explanatory levels vary, all address the AIP model to some extent and share the viewpoint that BL/DAS alone do not explain the totality of EMDR treatment effects. In other words, they share the ambition of elaborating on the theoretical underpinnings of the AIP model and of EMDR as a comprehensive psychotherapy approach.

Of the seven models, four focus principally on psychological processes, two exclusively (the Dialectical and GDPP perspectives) and two referring to speculatively corroborative neural evidence to support the purported psychological mechanisms (Zeigarnik and BPS-AIP). Not surprisingly, the two models that are primarily neural, the TnC and the 3DMES, were proposed by neuroscience researchers. The NBMTR adopts an intermediary position and addresses both the neural aspects (based on the large-scale neural networks) and the psychological dimension (with the predictive processing theory).

According to Shapiro’s AIP model, psychopathology and personality development can be explained as the consequences of adverse or disturbing life experiences (sometimes referred to as *big T* and *small t* traumas). While initial research focused on PTSD, subsequent studies have offered promising support in favor of positive outcomes for other forms of mental disorders (28). Shapiro herself consistently described trauma in the broadest sense as related to the blocked processing of memories associated with these experiences, thus generating dysfunction. Among the complementary models, the Zeignarik and BPS-AIP proposals join Shapiro’s understanding of trauma (traumatic experiences or traumatic memories) as the common origin of all psychopathology; so do the Dialectical and NBMTR hypotheses, while relying more narrowly on the example of PTSD. The TnC, 3DMES, and GDPP perspective exclusively address the formation of PTSD.

As for the popular notion that traumatic memories are static and frozen-in-time, remaining in their original state-specific form, most theoretical models either agree (TnC, NBMTR) or do not address the topic. The GDPP perspective stands apart in its disagreement, considering that the mental system will inevitably attempt to update memories and integrate them into current belief networks. Instead, the GDPP model explores the reasons why some individuals may be predisposed to avoiding situations that would generate prediction errors and the updating of trauma-related beliefs.

Apart from the Dialectical perspective and the 3DMES, all complementary theoretical models refer to predictions or predictive processing. While most present these as compatible with the concept of associative linking of maladaptively stored memories with adaptive memory networks, the GDPP perspective once again distinguishes itself by taking a different stance, going as far as to advocate for a significant modification to the AIP model, replacing the notion of associative processing with that of predictive processing: the mere facilitation of associations would not be sufficient for therapeutic change in this view. Rather, what is required is the updating of beliefs generated by prediction errors that promote integration of traumatic information with adaptive information that is sufficiently consistent with the individual’s belief network. Overall, however, there appears to be a majority opinion in favor of propositional and predictive processing theories (e.g., in relation to GSAs, IWMs, belief modules, attention schemas, etc.) whether they are explicitly mentioned or not.

Another recurring notion among the complementary proposals is that of balance necessary to health (e.g., within or between neural networks, between dialectical movements, between attention to the inner experience and awareness of the outer world), with the paired idea that pathology arises when this equilibrium is broken. This dynamic view is in contrast with the immobility depicted in the AIP model’s blocked processing and static memories.

Some questions appear to remain unanswered by these complementary theoretical models, beyond the novel insights, agreements, and areas of dispute they bring about. If all psychopathology is trauma-based (stemming from disturbing or adverse life experiences), why is there such a diverse range of mental disorders and dysfunction in addition to PTSD, and what are the specific determinants of each? There is yet to be provided a theoretical model of EMDR therapy that explains why, following similar experiences and comparable backgrounds, one individual will develop PTSD, a second will develop a different mental disorder, and a third may remain healthy or subclinical.

In addition, even if one admits that all non-organically based psychopathology stems from trauma (which remains to receive a precise definition), that does not mean that treatment strategies or effects for PTSD necessarily apply to other diagnoses with different symptomologies. Why would mechanisms of EMDR therapy at play in the treatment of PTSD be meaningful with other disorders or forms of dysfunction? On the other hand, if the same mechanisms are involved for all disorders in all contexts, is there truly a need for the high number of special EMDR protocols that continue to be developed ever since Shapiro’s (7) initial *protocols and procedures for special situations*?

In fact, if the AIP system is innate and universal, if all psychotherapeutic change is related to accelerated information processing, and if all psychopathology is related to pathogenic memories (29), then that effectively confirms Shapiro’s stance that the AIP model could serve as a unifying theory for all psychological modalities. In this sense, the AIP model becomes a meta-model describing the common factors for all psychotherapeutic change and for all psychopathology (that is not organic or chemically induced).

In addition, EMDR therapy is viewed as an integrative, comprehensive psychotherapy approach that cannot be reduced to the effects of its most recognizable component, inherent in its name: the eye movements (or other BL/DAS). Recently, the *What is EMDR?* workgroup of the Council of Scholars stated that what makes EMDR therapy unique is the way in which its procedural elements are brought together (3). But in what way is this aggregation of integrative elements unique to EMDR therapy and possibly superior to other modalities? None of the extant theoretical models offer any guidance on this issue, beyond the specific effects of BL/DAS, most often eye movements—with the outstanding question of whether hypotheses based on eye saccades possess any relevance to the practice of EMDR therapy in clinical practice.

In summary, most complementary theoretical models are exclusively or largely psychological, while the others introduce novel ideas at the neural level. All focus on the effects of EMDR therapy as a comprehensive psychotherapy approach with its principles, procedures, and protocols, while sometimes elaborating on the specific role of BL/DAS. Many refer to the notion of balance and most include concepts related to propositional theories or predictive processing.

It should be noted that the reviewed theoretical models did not recommend any modifications to current treatment protocols that are not compatible with, or already documented (in comparable language) within, the standard protocol and procedures, beyond those that are already well accepted within the scope of recognized special protocols.

Overall, the cumulative contribution of these proposals does appear to bolster Shapiro’s original AIP model, potentially offering additional depth and breadth to case conceptualization and treatment planning in clinical practice, as well as a more precise theoretical understanding, but without involving any significant changes to current standard clinical practice.

The current review possesses the limitations of all narrative reviews, in that it cannot claim the methodology that is associated with scoping or systematic reviews. While it is possible that some theoretical proposals, complementary to the AIP model, were omitted, the present paper should be viewed as a preliminary study exploring the potential merit and interest of the subject matter. Furthermore, a detailed review of the literature of empirical studies in relation to the hypotheses of each theoretical model would have allowed for a more in-depth critical analysis of each model, but such an endeavor is beyond the scope of the present manuscript and the space restrictions of a single article.

Future studies should evaluate the role of associative versus predictive processing in EMDR therapy, verify the compatibility of the neurological and psychological processes described within the various models, and determine whether explanatory hypotheses based on saccadic eye movements are at all relevant to theoretical models and or to models of mechanisms of action, given the observation that the “smooth eye pursuit that occurs during [bilateral stimulation] in EMDR therapy is actually very different from … saccadic movements” (2, p. 15). A larger, more comprehensive, and systematic review might address both hypotheses regarding mechanisms of action related to the different mediators of EMDR therapy, including but not limited to BL/DAS, and broader theoretical proposals regarding EMDR therapy as a comprehensive psychotherapy approach. Future theoretical papers, on the other hand, may contrast the currently described theoretical proposals for EMDR therapy and its AIP model with the models underlying other psychotherapy approaches and models of trauma-related and general psychopathology. Finally, future endeavors may aim to assess how the AIP model might incorporate the unique determinants and trajectories that lead to the development of different forms of trauma-related psychopathology, beyond PTSD.

## Data availability statement

The original contributions presented in the study are included in the article/supplementary material. Further inquiries can be directed to the corresponding author.

## Author contributions

JR: Writing – review & editing, Writing – original draft, Methodology, Conceptualization. LV: Writing – review & editing, Investigation. CT: Writing – review & editing, Supervision.
